# Depletion of Homeostatic Antibodies against Malondialdehyde-Modified Low-Density Lipoprotein Correlates with Adverse Events in Major Vascular Surgery

**DOI:** 10.3390/antiox11020271

**Published:** 2022-01-29

**Authors:** Adam Hartley, Magapu Pradeep, Victor Van den Berg, Ameer Hamid Ahmed Khan, Hasan Ali Shah, Mohammed Allaf, Anna Chow, Mikhail Caga-Anan, Joseph Shalhoub, Wolfgang Koenig, Michael Fisher, Dorian O. Haskard, Ramzi Y. Khamis

**Affiliations:** 1 National Heart and Lung Institute, Imperial College London, London W12 0NN, UK; adam.hartley12@imperial.ac.uk (A.H.); m.caga-anan@imperial.ac.uk (M.C.-A.); d.haskard@imperial.ac.uk (D.O.H.); 2 Liverpool Heart and Chest Hospital, Liverpool L14 3PE, UK; pradeep.magapu@lhch.nhs.uk; 3 Department of Cardiology, Erasmus Medical Centre, University Medical Center Rotterdam, 3015 GD Rotterdam, The Netherlands; v.vandenberg@erasmusmc.nl; 4 Department of Medicine, Imperial College London, London SW7 2AZ, UK; ameer.khan1@nhs.net (A.H.A.K.); hasan_a_shah@yahoo.co.uk (H.A.S.); mohammed.allaf@nhs.net (M.A.); anna.chow2@nhs.net (A.C.); 5 Academic Section of Vascular Surgery, Department of Surgery & Cancer, Imperial College London, London, UK and Imperial Vascular Unit, Imperial College Healthcare NHS Trust, London W2 1NY, UK; j.shalhoub@imperial.ac.uk; 6 Deutsches Herzzentrum München, Technische Universität München, Munich, Germany and DZHK (German Centre for Cardiovascular Research), Partner Site Munich Heart Alliance, Lazarettstraße 36, 80636 Munich, Germany; koenig@dhm.mhn.de; 7 Institute for Cardiovascular Medicine and Science, Liverpool Heart and Chest Hospital NHS Trust and Royal Liverpool and Broadgreen NHS Trust, Liverpool L14 3PE, UK; michael.fisher@liverpoolft.nhs.uk

**Keywords:** atherosclerosis, low-density lipoprotein (LDL), oxidized LDL, complement, anti-oxidized LDL antibodies, immune complex

## Abstract

We aimed to investigate if major vascular surgery induces LDL oxidation, and whether circulating antibodies against malondialdehyde-modified LDL (MDA-LDL) alter dynamically in this setting. We also questioned relationships between these biomarkers and post-operative cardiovascular events. Major surgery can induce an oxidative stress response. However, the role of the humoral immune system in clearance of oxidized LDL following such an insult is unknown. Plasma samples were obtained from a prospective cohort of 131 patients undergoing major non-cardiac vascular surgery, with samples obtained preoperatively and at 24- and 72 h postoperatively. Enzyme-linked immunoassays were developed to assess MDA-LDL-related antibodies and complexes. Adverse events were myocardial infarction (primary outcome), and a composite of unstable angina, stroke and all-cause mortality (secondary outcome). MDA-LDL significantly increased at 24 h post-operatively (*p* < 0.0001). Conversely, levels of IgG and IgM anti-MDA-LDL, as well as IgG/IgM-MDA-LDL complexes and total IgG/IgM, were significantly lower at 24 h (each *p* < 0.0001). A smaller decrease in IgG anti-MDA-LDL related to combined clinical adverse events in a post hoc analysis, withstanding adjustment for age, sex, and total IgG (OR 0.13, 95% CI [0.03–0.5], *p* < 0.001; *p* value for trend <0.001). Major vascular surgery resulted in an increase in plasma MDA-LDL, in parallel with a decrease in antibody/complex levels, likely due to antibody binding and subsequent removal from the circulation. Our study provides novel insight into the role of the immune system during the oxidative stress of major surgery, and suggests a homeostatic clearance role for IgG antibodies, with greater reduction relating to downstream adverse events.

## 1. Introduction

Low-density lipoprotein (LDL) readily undergoes chemical modification leading to the formation of malondialdehyde (MDA)-LDL and other peroxidation products that have a variety of toxic effects on endothelial and other cells [1]. Under homeostatic conditions, the humoral immune system is involved in the protective clearance of these and other oxidatively modified proteins. Natural antibodies and complement act in concert to perform this role, removing cellular debris to the liver and lymphoid organs. IgM and IgG antibodies targeting MDA-LDL are a significant component of the antibody repertoire that reacts with modified LDL [2]. Higher circulating levels of IgM anti-MDA-LDL have been generally found to confer protection from cardiovascular events [3], but the prognostic relevance of IgG anti-MDA-LDL levels with disease states is less clear [4,5,6]. Circulating antibodies can form complexes with modified LDL, and levels of IgM-LDL complexes are also postulated to be protective [7,8,9,10]. IgG-LDL complexes, on the other hand, may be detrimental [5,11]. Complement components may also have both beneficial and harmful effects within the vessel wall. On the one hand, the classical and lectin pathways seem to be protective, in part by enhancing modified LDL clearance [12]. On the other hand, the alternative pathway and terminal complement complexes are potentially more dysfunctional and proinflammatory [13].

Major surgery can induce a stress response and lead to activation of oxidation and inflammatory pathways [14]. Intraoperative ischaemia/reperfusion phases that occur during vascular surgery, especially in the treatment of occlusive peripheral arterial disease or with cross-clamping during aortic procedures, augment free radical generation, amplifying oxidative stress [15,16]. Unsurprisingly, patients undergoing major vascular surgery are at significant risk of perioperative cardiovascular sequalae, including acute coronary syndromes, stroke, and death [17].

We have developed a set of enzyme-linked immunosorbent assays (ELISAs) for measuring MDA-LDL, anti-MDA-LDL antibodies, and MDA-LDL immune complexes, and in this study we have applied them to assess how the humoral immune system is involved in MDA-LDL clearance following the challenge of vascular surgery. We have based our assays for MDA-LDL on monoclonal antibody LO1, an anti-MDA-LDL autoantibody derived from an LDL-receptor deficient mouse [18]. Using these assays, we have been able to demonstrate reciprocal changes in plasma MDA-LDL and anti-MDA-LDL antibodies, suggesting antibody utilization and depletion in response to surgical stress. In addition, we show that dynamic changes in IgG anti-MDA-LDL are linked to adverse clinical outcomes.

## 2. Methods

### 2.1. Ethical Approval

Ethical approval for the study was granted by the local research ethics committee (North West National Research Ethics Service) prior to commencement (REC reference 11/NW/0767). All patients gave written informed consent and the investigation conformed to the principles outlined in the Declaration of Helsinki.

### 2.2. Study Design

The Stress Induced Myocardial Infarction After Non-cardiac vascular surgery (SIMIAN) study was a prospective observational study conducted at the Royal Liverpool University Hospital, Liverpool, UK. Inclusion criteria were restricted to those aged 18 years old or over undergoing elective vascular surgery (including open and endovascular abdominal aortic aneurysm repair, vascular arterial bypass, and limb amputation). Exclusion criteria were inability or unwillingness to give informed consent, clinical evidence of an acute infection at the time of preoperative assessment, acute coronary syndrome within the preceding three months, or inclusion in other clinical studies.

The primary endpoint was defined as myocardial infarction, based upon a rise in high sensitivity troponin T to >60 ng/L, measured when clinically indicated, as well as symptoms of ischaemia or new electrocardiogram changes, including the development of pathological Q-waves. The secondary endpoint was defined as a composite of unstable angina, stroke and all-cause mortality (Supplementary Table S1). All endpoints were assessed post-operatively until discharge from hospital.

### 2.3. Blood Samples

Preoperative blood samples were obtained 12–24 h prior to surgery and postoperative samples at 24 and 72 h after surgery. Blood was anticoagulated with 3.2% trisodium citrate, after which blood was centrifuged at 2000× *g* for 15 min to separate plasma from formed elements including platelets. Plasma samples were stored at −80 °C prior to thawing to room temperature before use. The platelet-poor plasma was used for ELISAs.

### 2.4. Generation of MDA-LDL

Native LDL was modified via conjugation with MDA to form MDA-LDL as previously described by Palinski et al. [19]. Briefly, MDA was prepared via acid hydrolysis of malondialdehyde bis (dimethyl acetal) (Sigma-Aldrich, Poole, UK). 0.5 M MDA solution was then incubated with native LDL at 37 °C for 3 h, to produce MDA-LDL. Following generation, MDA-LDL was eluted through a Zeba Spin desalting column (ThermoFisher Scientific, Waltham, MA, USA) with PBS and 0.01% ethylenediaminetetraacetic acid added to prevent further oxidation.

### 2.5. ELISA to Detect MDA-LDL and Apolipoprotein B-100 (ApoB)

Sandwich ELISA protocols were established to measure the majority of components within our study. MDA-LDL was measured using LO1 (10 μg/mL) as the capture antibody for MDA-LDL [18], whilst polyclonal goat anti-human anti-ApoB (Abcam, Cambridge, MA, USA; 1:2000 dilution) was used as the capture antibody for ApoB. A biotinylated anti-ApoB antibody (Abcam, Cambridge, MA, USA; 1:2000), followed by horseradish peroxidase (HRP)-conjugated streptavidin (R&D Systems, Minneapolis, MN, USA; 1:200) were used for detection of both MDA-LDL and ApoB. For these assays, and all other ELISAs, development was achieved by adding 3,3′,5,5′-tetramethylbenzidine (Sigma Aldrich, Poole, UK), after which the reaction was stopped with 0.5 M H_2_SO_4_. Plates were read at an optical density of 450 nm using a Synergy HT microplate reader (BioTek, VT, USA).

### 2.6. ELISA to Detect Antibodies to MDA-LDL

MDA-LDL at a concentration of 10 µg/mL was used on the solid phase to quantify anti-MDA-LDL antibodies in plasma diluted 1:80 and 1:120 for IgG and IgM, respectively, as previously reported [3]. Detection antibodies were either an unlabeled mouse anti-human IgG (Southern Biotech, Birmingham, AL, USA) followed by HRP-conjugated polyclonal rabbit anti-mouse antibody (Dako, Cambridgeshire, UK) (1:2000) or biotinylated mouse anti-human IgM (Southern Biotech, Birmingham, AL, USA) followed by HRP-conjugated streptavidin (1:200).

### 2.7. ELISA to Detect Total IgG and IgM Antibodies

Total IgG and IgM antibody levels were measured in sandwich format. Goat anti-human IgG (1:800) or mouse anti-human IgM (1/400) (Southern Biotech, Birmingham, AL, USA) were used for capture. Biotinylated goat F(ab’)^2^ anti-human IgG (1/50,000) or biotinylated mouse anti-human IgM (1/15,000) (Southern Biotech, Birmingham, AL, USA), followed by HRP-conjugated streptavidin (1:200), were used for detection.

### 2.8. ELISA to Detect C3

Polyclonal chicken anti-human anti-C3 (Sigma Aldrich, Poole, UK; 1:4000) was used as the capture antibody for total C3. The sample dilution was 1:40. A biotinylated monoclonal mouse anti-human anti-C3 antibody (Biolegend, San Diego, CA, USA; 1:1000) followed by HRP-conjugated streptavidin as above was used for detection.

### 2.9. ELISA to Detect IgG/MDA-LDL, IgM/MDA-LDL and C3/MDA-LDL Complexes

LO1 at a concentration of 10 µg/mL was used as the capture antibody for all MDA-LDL complex ELISA assays. The plasma sample dilution was 1:40. Detection was with biotinylated mouse anti-human IgG (Southern Biotech, Birmingham, AL, USA; 1:5000), biotinylated mouse anti-human IgM (Southern Biotech, Birmingham, AL, USA; 1:2000), or monoclonal mouse anti-human anti-C3 antibody (Biolegend, San Diego, CA, USA; 1:2000) for IgG, IgM, and C3 complexes, respectively.

For MDA-LDL, ApoB, and total antibody levels, log transformed standard curve optical density values were fitted, using non-linear regression onto a sigmoidal, four-parametric logistic curve. This was then used to interpolate sample concentrations from sample optical density (OD) values. For other assays, raw OD values were analyzed.

### 2.10. Statistical Analysis

Normality of the data were assessed with the D’Agostino Pearson omnibus normality test. For non-normally distributed data, paired data were assessed using the Wilcoxon matched-pairs signed rank test and unpaired data were assessed using the Mann–Whitney test. Continuous variables are presented as mean ± standard deviation (SD) or median with interquartile range (IQR), depending on the normality of the distribution. Categorical variables are presented as numbers with percentages. The correlation of antibodies against MDA-LDL and total IgG/IgM antibodies was assessed using Spearman’s rank correlation coefficient.

Statistical analysis was performed using R version 3.3.1 (R Foundation for Statistical Computing, Vienna, Austria). Using logistic regression, we assessed the relationship between the biomarkers and combined clinical endpoints in a post hoc analysis, both for the baseline measurement and the change or delta between the baseline measurement and the 24 h measurements. We started with evaluating all biomarkers as continuous variables and assessed the relationship between the biomarkers and the clinical endpoints by an increase of one standard deviation for the baseline measurements, and by percentage change for the 24 h delta measurements. Hereafter, we divided the baseline concentrations and 24 h deltas in tertiles (smallest to largest increase or largest to smallest decrease, depending on the dynamics of the biomarker) and repeated the same analysis; the first tertile was used as a reference. All analyses were adjusted for age and sex (Model 1). The change at 24 h in anti-MDA-LDL antibodies was further adjusted, with the inclusion of change in total IgG/IgM, as appropriate (Model 2). Statistical significance was defined as a two-tailed *p*-value < 0.05.

### 2.11. Role of the Funding Sources

The study itself was not sponsored, and funding sources had no role in study design or data collection or analysis.

## 3. Results

### 3.1. Baseline Characteristics

Between January 2012 and January 2014, 131 patients were recruited into the study (Table 1). The median age was 71.5 (interquartile range 65–77) years and 80.2% were male. The most performed surgeries were endovascular abdominal aortic aneurysm repair and arterial bypass, accounting for over 90% of procedures. There was a high prevalence of cardiovascular risk factors, as would be expected. Thus, 91.6% were current or ex-smokers, 74% had hypertension, and 21.4% had diabetes mellitus.

### 3.2. Temporal Changes in Biomarkers

To correct for changes in plasma LDL concentration due to intraoperative blood loss, fluid shifts, and dilution, each MDA-LDL concentration was expressed as a ratio to ApoB concentration. The adjusted MDA-LDL levels were then used for evaluation of the dynamics of MDA-LDL, and a similar adjustment was performed for complexes of IgG, IgM, and C3 with MDA-LDL.

**Figure 1:**
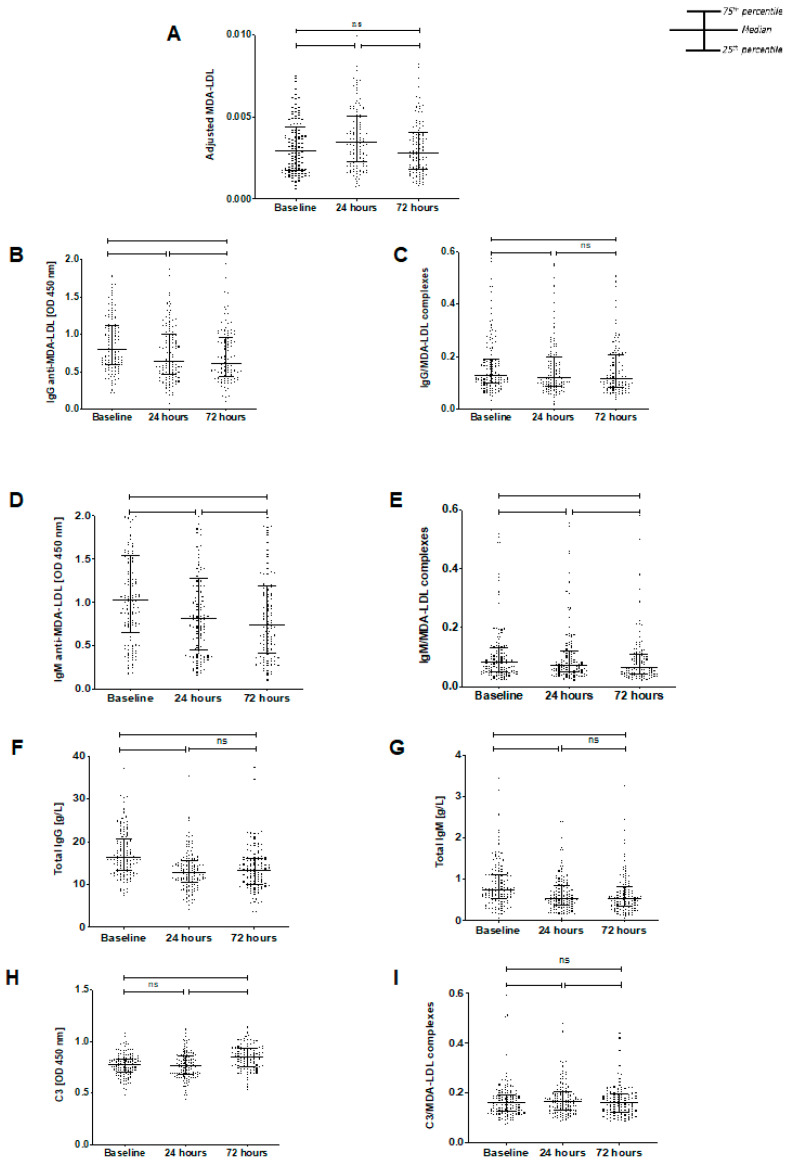
The perioperative temporal changes in assessed biomarkers in 131 patients in the Stress Induced Myocardial Infarction After Non-cardiac vascular surgery (SIMIAN) study. (**A**) MDA-LDL increases from baseline at 24 h (*p* < 0.0001) and subsequently decreases at 72 h (*p* < 0.0001). There was no significant difference between baseline and 72 h (*p* = 0.3290). (**B**) IgG anti-MDA-LDL significantly decreased at 24 h post-surgery and declined further at 72 h post-surgery (*p* < 0.0001, *p* = 0.0017, respectively and *p* < 0.0001 between baseline and 72-h). (**C**) IgG/MDA-LDL complex levels declined at 24 h postoperatively (*p* < 0.0001) and did not change significantly at 72 h (*p* = 0.0937, *p* < 0.0001 vs. baseline). (**D**) IgM anti-MDA-LDL levels significantly declined between baseline and 24 h postoperatively (*p* < 0.0001). Levels then continued to reduce between 24 h and 72 h (*p* = 0.0243, *p* < 0.0001 vs. baseline). (**E**) IgM/MDA-LDL complex levels significantly decreased at 24 h postoperatively (*p* < 0.0001) and then decreased further at 72 h (*p* = 0.0149, *p* < 0.0001 vs. baseline). (**F**) Total IgG decreased at 24 h (median of differences −3.841, *p* < 0.0001) and then remained stable (median of differences −0.165, *p* = 0.824). Overall, there was a significant decrease (median of differences −3.722, *p* < 0.0001). (**G**) Similarly, total IgM decreased from baseline to 24 h post-operatively (median of differences −0.17, *p* < 0.0001), then did not change significantly. There was an overall decline from basline to 72 h (median of differences −0.17, *p* < 0.0001). (**H**) Total C3 did not change at 24 h (*p* = 0.0968), but then significantly increased from 24- to 72 h (*p* < 0.0001 from baseline and from 24-h). (**I**) C3-oxLDL/ApoB complex levels significantly rose between baseline and 24 h (*p* = 0.0097) followed by a decline between 24 and 72 h (*p* < 0.0001, *p* = 0.2603 vs. baseline). ns: non-significant, *: *p* ≤ 0.05, **: *p* < 0.01, ****: *p* < 0.0001. Wilcoxon matched-pairs signed rank test used to assess significance, *p* ≤ 0.05.

**MDA-LDL** Adjusted MDA-LDL levels significantly increased between baseline and 24 h (median of differences 0.0004, *p* < 0.0001) but then reduced to pre-operative baseline levels by 72 h (median of differences −0.0005, *p* < 0.0001) (Figure 1A). There was no significant difference between levels at baseline and 72 h (*p* = 0.329).

**IgG anti-MDA-LDL antibodies and complexes** IgG anti-MDA-LDL levels significantly reduced between baseline and 24 h postoperatively (median of differences −0.1215, *p* < 0.0001) and continued to decline between 24 and 72 h (median of differences −0.0268, *p* = 0.0017) (Figure 1B). Likewise, IgG/MDA-LDL complex levels significantly reduced at 24 h postoperatively (median of differences −0.0123, *p* < 0.0001) and remained reduced at 72 h (Figure 1C).

**IgM anti-MDA-LDL antibodies and complexes** IgM anti-MDA-LDL levels also significantly declined between baseline and 24 h postoperatively (median of differences–0.1613, *p* < 0.0001) (Figure 1D), and then declined further between 24 h and 72 h (median of differences−0.017, *p* = 0.0243). Similarly, IgM/MDA-LDL complexes were significantly decreased at 24 h postoperatively (median of differences −0.0062, *p* < 0.0001), and were decreased further at 72 h (median of differences −0.0025, *p* = 0.0149) (Figure 1E).

**Total IgG immunoglobulins** Total IgG antibody levels decreased significantly from baseline to 24 h (median of differences −3.841g/L, *p* < 0.0001) (Figure 1F) and then stabilized at 72 h (median of differences −0.165g/L, *p* = 0.824). Overall, from baseline to 72 h there was a significant decrease (median of differences −3.722g/L, *p* < 0.0001).

**Total IgM immunoglobulins** Total IgM antibodies also decreased from baseline to 24 h post-operatively (median of differences −0.17g/L, *p* < 0.0001) (Figure 1G). Levels then remained steady, with an overall significant decline from baseline to 72 h (median of differences −0.17g/L, *p* < 0.0001).

**Complement C3 and complexes** In contrast to IgG or IgM anti-MDA-LDL or total IgG and IgM immunoglobulins, C3 and C3 complexes were unchanged postoperatively at 24 h (median of difference −0.0269, *p* = 0.0968) (Figure 1H). However, there was then a significant increase in C3 at 72 h (from baseline, median of differences 0.0572, *p* < 0.0001; from 24-h, median of differences 0.0783, *p* < 0.0001). C3/MDA-LDL complexes followed the same course as MDA-LDL, significantly increasing between baseline and 24 h (median of differences 0.0056, *p* = 0.0097) and then declining between 24- and 72 h (median of differences −0.0071, *p* < 0.0001). There was no significant difference between baseline and 72 h (*p* = 0.2603) (Figure 1I).

### 3.3. Antibody Relationships

Basal levels of IgG and IgM anti-MDA-LDL did not correlate (r = 0.166, *p* = 0.0557), but the changes at 24 h were strongly correlated (r = 0.431, *p* < 0.0001). Similarly, basal levels of total IgG and IgM were only weakly correlated (r = 0.189, *p* = 0.03), whilst the 24 h changes were strongly positively related (r = 0.602, *p* < 0.0001). Baseline and 24 h delta levels of IgG and IgM anti-MDA-LDL correlated with total IgG and IgM, respectively (r = 0.377 and r = 0.477 for baseline and delta IgG; r = 0.633 and r = 0.486 for baseline and delta IgM, all *p* < 0.0001).

### 3.4. Relationships between Biomarkers and Clinical Events

A myocardial infarction, the primary endpoint, occurred in nine patients, whilst the composite secondary endpoint occurred in 16 patients. The secondary endpoint comprised 14 patients with unstable angina, none with strokes and two deaths. In a post hoc analysis, given the low occurrence of the primary endpoint, the 25 patients with a primary or secondary endpoint (thus comprising myocardial infarction [9], unstable angina [14], and mortality [2]) were combined and compared against those experiencing no endpoints (106 patients).

Basal levels of IgG anti-MDA-LDL antibodies did not relate to clinical events after adjustment for age and sex (Model 1) (Supplementary Table S2). However, the decrease from baseline to 24 h postoperatively was strongly related to clinical events (smallest decrease odds ratio [OR] 0.13, 95% confidence interval [CI] [0.03–0.5], *p* < 0.001; *p* value for trend <0.001; *p* value per percentage change <0.001) (Table 2). The temporal change in total IgG was also associated with clinical events (smallest decrease tertile OR 0.36, 95% CI [0.12–1.04], *p* = 0.06; *p* value for trend 0.04; *p* value per percentage change 0.02) (Table 3). Therefore, we further scrutinized the relationship with IgG anti-MDA-LDL by adjusting for the change in total IgG, in addition to age and sex (Model 2). The relationship between 24 h change in IgG anti-MDA-LDL and clinical events remained (smallest decrease tertile OR 0.16, 95% CI [0.04–0.66], *p* = 0.01, *p* = 0.008 for trend, *p* = 0.03 per percentage increase).

Similarly, basal levels of IgM anti-MDA-LDL did not relate to clinical events (Supplementary Table S2), but the 24 h decrease was associated (smallest decrease tertile OR 0.21, 95% CI [0.06–0.71], *p* = 0.01, *p* = 0.01 for trend, *p* = 0.01 per percentage increase) (Table 2). The dynamic change in total IgM was also related to clinical events (smallest decrease OR 0.15, 95% CI [0.04–0.56], *p* < 0.001, *p* < 0.001 for trend, *p* < 0.001 per percentage increase) (Table 3). After adjustment of IgM anti-MDA-LDL with total IgM in Model 2, the relationship with clinical events was lost (smallest decrease OR 0.41, 95% CI [0.1–1.71], *p* = 0.22, *p* = 0.25 for trend, *p* = 0.25 per percentage increase).

There were no relationships between the other biomarkers and clinical events, except for C3 at baseline and the 24 h increase in C3/MDA-LDL complexes (Supplementary Table S3). The middle tertile of C3 related to clinical events (OR 3.67, 95% CI 1.05–12.82, *p* = 0.04), whist there was an apparent trend with the highest tertile, albeit with a wide confidence interval (OR 3.11, 95% CI 0.89–10.88, *p* = 0.08). A larger increase in C3/MDA-LDL related to clinical events with OR 4.19 (95% CI 1.35–12.97) *p* = 0.01, with *p* = 0.01 for trend as well as per percentage change).

## 4. Discussion

Our study shows that major non-cardiac vascular surgery results in significant dynamic changes in levels of MDA-LDL, as well as in components of the immune system involved in its clearance. MDA-LDL levels increased postoperatively and subsequently returned to baseline within 72-h. C3/MDA-LDL complexes followed a similar pattern, whilst total C3 was unchanged at 24 h and increased at 72 h. Conversely, IgG/IgM anti-MDA-LDL antibodies and their respective complexes significantly declined following surgery. Providing clinical significance to our laboratory observations, the magnitude of perioperative fall in IgG anti-MDA-LDL strongly related to cardiovascular endpoints, withstanding correction for age, sex, and total IgG levels.

The most significant finding of this study was that smaller reductions in anti-MDA-LDL antibodies, at 24 h postoperatively from preoperative baseline, correlated with a lower event rate (a composite of myocardial infarction, unstable angina, and mortality). This relationship was present for both IgG and IgM anti-MDA-LDL antibodies, albeit the correlation with IgM was lost after correction for total IgM levels. Further sufficiently powered studies to detect relationships with clinical events may also confirm the association with IgM anti-MDA-LDL. Accordingly, a greater decrease in anti-MDA-LDL antibodies (i.e., more utilization and consequent clearance) was associated with adverse events. It is likely that the anti-MDA-LDL antibodies are binding to, and neutralizing, MDA-LDL that is produced by oxidant stress of major surgery in a homeostatic process. One suggested mechanism for this relationship is that with greater oxidant stress, there is greater MDA-LDL production and accordingly greater IgG/IgM binding to MDA-LDL, forming complexes which are then cleared from the bloodstream via the reticuloendothelial system for excretion (with a reduction in the measured IgG/IgM anti-MDA-LDL antibody levels). However, it does remain unclear whether the oxidative stress milieu is the primary trigger of the clinical event (for example, with plaque rupture and subsequent vessel occlusion, leading to a Type 1 or 2 myocardial infarction), or if oxidative stress is secondary to the event itself (for example a consequence of ischaemic myocardium). Either way, these findings suggest that greater utilization of anti-MDA-LDL antibodies following a large systemic insult relates to adverse consequences.

We have recently shown that major cardiac surgery with coronary artery bypass grafting results in a depletion of both IgG and IgM anti-MDA-LDL antibodies in tandem with an increase in MDA-LDL levels [18]. In other earlier studies, transient increases in plasma oxidized LDL and reduction in plasma anti-MDA-LDL antibodies have been identified following percutaneous coronary intervention, albeit over a shorter time course [20]. Other than these studies, there are few data on how modified LDL and anti-modified LDL antibodies change dynamically in response to surgical interventions. Atherosclerotic plaque rupture with release of necrotic core contents is not a likely cause for the oxLDL increases seen in this study, however. The most likely explanation for our findings is that oxidation of circulating LDL is a consequence of surgery-related systemic oxidative stress.

IgM and IgG total and anti-MDA-LDL antibody levels declined postoperatively in this study, most likely attributable to a consumptive process, with removal of IgM/MDA-LDL and IgG/MDA-LDL complexes to the reticuloendothelial system. Interestingly, anti-MDA-LDL antibodies continued to decline from 24 to 72 h, whereas total antibodies remained constant. This may possibly relate to persisting oxidative stress with selective antibody depletion. Although circulating MDA-LDL levels rose, both IgG and IgM/MDA-LDL complexes fell in parallel with the respective antibody levels, suggesting that antibody binding and clearance occurs, and that supply fails to keep up with utilization. That this was not the case for C3 and C3/MDA-LDL complexes can be explained by C3 being synthesized and released into the circulation as an acute phase reactant, in response to cytokines and other inflammatory stimuli [21]. Interestingly, greater increases in C3/MDA-LDL at 24 h post-operatively related to cardiovascular events in this study. These findings suggest that greater inflammation, combined with greater lipoprotein oxidation in response to the surgical insult, relates to worse clinical outcomes. However, C3/MDA-LDL complexes themselves may not be solely proinflammatory, as they may remain under regulatory control by both complement factor H and decay accelerating factor in plasma, preventing progress beyond the level of C3 in the complement cascade [22,23].

The overall conclusion to be drawn from these findings is that the immunological means for removing modified LDL from the circulation in an acute stress situation may be limited. This could be important, as modified LDL has the capacity to promote dysfunction or activation of vascular endothelial and other cells [24].

The main limitations from this study lie in the lower-than-expected primary event rate in the study; as such, it is possible that associations between biomarkers and clinical events are under-detected. A post hoc analysis was therefore performed using combined primary and secondary endpoints in an attempt to address this; however, this is a less specific cardiovascular outcome than myocardial infarction alone. Furthermore, the fixed time frames of the plasma samples provided in this study make it difficult to elucidate exactly when plasma levels peak and when they are cleared. Future studies could provide more frequent plasma samples over a longer period to better assess this.

## 5. Conclusions

Our analysis in the acute setting of major vascular surgery reveals several findings and propositions. Firstly, MDA-LDL levels are increased in response to the surgical intervention. Secondly, there is consumptive depletion of IgG and IgM antibodies involved in antigen clearance as MDA-LDL complexes. The failure of antibody supply to keep up with demand results in uncomplexed MDA-LDL with potential deleterious effects on vascular endothelial and other cells. Thirdly, the magnitude of IgG anti-MDA-LDL antibody depletion correlates inversely with adverse cardiovascular outcomes in this setting, as smaller temporal reductions at 24 h related to less occurrence of myocardial infarction, unstable angina and mortality. These novel assays have the potential for clinical use as biomarkers of lipoprotein immune handling and for risk-stratification for the prediction of post-operative cardiovascular events.

## Figures and Tables

**Table 1:** Baseline patient characteristics of the 131 patients in the Stress Induced Myocardial Infarction After Non-cardiac vascular surgery (SIMIAN) study. AAA: abdominal aortic aneurysm, CABG: coronary artery bypass grafting, CKD: chronic kidney disease, COPD: chronic obstructive pulmonary disease, CVA: cerebrovascular accident, GORD: gastro-oesophageal reflux disease, IHD: ischaemic heart disease, MI: myocardial infarction, TB: tuberculosis, TIA: transient ischaemic attack.

SIMIAN Study Baseline Characteristics	
Characteristic	Study Population (*n* = 131)
**Age**, **years** (median, IQR)	71.5 (65–77)
**Sex**	
Male	105 (80.2%)
Female	26 (19.8%)
**Ethnicity**	
White	130 (99.2%)
Asian	1 (0.8%)
**Hypertension**	97 (74.0%)
**IHD**	35 (26.7%)
**Previous MI**	34 (26.0%)
**Previous CABG**	16 (12.2%)
**Diabetes**	28 (21.4%)
**CKD Stages**	
I	51 (38.9%)
II	48 (36.6%)
III	29 (22.1%)
IV	3 (2.3%)
**Previous CVA**	12 (9.2%)
**Previous TIA**	12 (9.2%)
**Smoking status**	
Current smoker	42 (32.1%)
Ex-smoker	78 (59.5%)
Non-smoker	11 (8.4%)
**Operation**	
Open AAA	4 (3.1%)
Endovascular AAA	61 (46.6%)
Arterial Bypass	58 (44.3%)
Limb Amputation	7 (5.3%)
Unavailable Information	1 (0.8%)
**Other co-morbidities**	
Neurological disease	4 (3.1%)
Rheumatological disease	28 (21.4%)
Malignancy	22 (16.8)
Asthma	6 (4.6%)
COPD	39 (29.8%)
TB	3 (2.3%)
GORD	32 (24.4%)
Peptic Ulcer Disease	12 (9.2%)
Hepatic Disease	3 (2.3%)

**Table 2:** Odds ratios of events (myocardial infarction, unstable angina or mortality) in relation to the dynamic changes in MDA-LDL, IgG/IgM anti-MDA-LDL antibodies and related complexes from baseline to 24-h. (Per SD increase in antibodies and in antibody tertiles). Model 1: adjusted for age and sex. Model 2: adjusted as in Model 1 with the addition of dynamic change in total IgG or IgM levels as appropriate.

		24-Hour Change			
		Model 1		Model 2	
		OR (95% CI)	*p* Value	OR (95% CI)	*p* Value
Per% change in MDA-LDL		0.99 (0.97–1.01)	0.48		
MDA-LDL	Smallest increase	1.00 (Ref.)			
	Mid	1.33 (0.46–3.8)	0.6		
	Largest increase	0.7 (0.22–2.26)	0.55		
Trend			0.57		
Per% change in IgG anti-MDA-LDL		0.94 (0.9–0.98)	**<0.001**	0.95 (0.9–0.99)	**0.03**
IgG anti-MDA-LDL	Largest decrease	1.00 (Ref.)		1.00 (Ref.)	
	Mid	0.23 (0.07–0.7)	**0.01**	0.26 (0.08–0.9)	**0.03**
	Smallest decrease	0.13 (0.03–0.5)	**<0.001**	0.16 (0.04–0.66)	**0.01**
Trend			**<0.001**		**0.008**
Per% change in IgM anti-MDA-LDL		0.96 (0.93–0.99)	**0.01**	0.98 (0.94–1.02)	0.25
IgM anti-MDA-LDL	Largest decrease	1.00 (Ref.)		1.00 (Ref.)	
	Mid	0.33 (0.11–0.96)	**0.04**	0.58 (0.16–2.08)	0.41
	Smallest decrease	0.21 (0.06–0.71)	**0.01**	0.41 (0.1–1.71)	0.22
Trend			**0.01**		0.25
Per% change in IgG/MDA-LDL complexes		0.99 (0.97–1.01)	0.49		
IgG/MDA-LDL complexes	Largest decrease	1.00 (Ref.)			
	Mid	0.2 (0.05–0.78)	**0.02**		
	Smallest decrease	0.73 (0.27–1.99)	0.54		
Trend			0.48		
Per% change in IgM/MDA-LDL complexes		0.99 (0.97–1.01)	0.24		
IgM/MDA-LDL complexes	Largest decrease	1.00 (Ref.)			
	Mid	0.34 (0.11–1.08)	0.07		
	Smallest decrease	0.52 (0.18–1.48)	0.22		
Trend			0.18		

**Table 3:** Odds ratios of events (myocardial infarction, unstable angina, or mortality) in relation to the dynamic changes in total IgG/IgM, C3, and C3/MDA-LDL complexes from baseline to 24-h. Model 1: adjusted for age and sex.

		24-Hour Change	
		Model 1	
		OR (95% CI)	*p* Value
Per% change in total IgG		0.97 (0.94–0.99)	**0.02**
Total IgG	Largest decrease	1.00 (Ref.)	
	Mid	0.29 (0.09–0.9)	**0.03**
	Smallest decrease	0.36 (0.12–1.04)	0.06
Trend			**0.04**
Per% change in total IgM		0.96 (0.93–0.99)	**<0.001**
Total IgM	Largest decrease	1.00 (Ref.)	
	Mid	0.37 (0.13–1.04)	0.06
	Smallest decrease	0.15 (0.04–0.56)	**<0.001**
Trend			**<0.001**
Per% change in complement C3		0.99 (0.96–1.03)	0.6
Complement C3	Smallest increase	1.00 (Ref.)	
	Mid	0.48 (0.16–1.46)	0.19
	Largest increase	0.59 (0.2–1.76)	0.35
Trend			0.32
Per% change in C3/MDA-LDL complexes		1.21 (1.04–1.41)	**0.01**
C3/MDA-LDL complexes	Smallest increase	1.00 (Ref.)	
	Mid	0.8 (0.2–3.26)	0.76
	Largest increase	4.19 (1.35–12.97)	**0.01**
Trend			**0.01**

## Data Availability

Fully anonymized data presented in this study can potentially be available upon specific request at the discretion of the corresponding author. The data are not publicly available for privacy and ethical reasons, given that the research participants who consented for the study did not provide specific consent to have their data shared in a public database.

## Supplementary Materials

The following supporting information can be downloaded at: https://www.mdpi.com/article/10.3390/antiox11020271/s1, Table S1: Primary and secondary endpoints of the Stress Induced Myocardial Infarction After Non-cardiac vascular surgery (SIMIAN) study, measured up to hospital discharge. ECG: electrocardiogram; Table S2: Odds ratios of events (myocardial infarction, unstable angina or mortality) in relation to baseline levels of MDA-LDL, IgG/IgM anti-MDA-LDL antibodies and related complexes. (Per SD increase in antibodies and in antibody tertiles). Model 1: adjusted for age and sex; Table S3: Odds ratios of events (myocardial infarction, unstable angina or mortality) in relation to baseline levels of total IgG/IgM, C3 and C3/MDA-LDL complexes. (Per SD increase in antibodies and in antibody tertiles). Model 1: adjusted for age and sex.

## Abbreviations

ApoB	apolipoprotein B-100
ELISA	enzyme-linked immunosorbent assay
HRP	horseradish peroxidase
LDL	low-density lipoprotein
MDA	malondialdehyde
MDA-LDL	malondialdehyde-conjugated LDL
OD	optical density
OxLDL	oxidized low-density lipoprotein
PBS	phosphate-buffered saline
SIMIAN	Stress Induced Myocardial Infarction After Non-cardiac vascular surgery
