# Supplementary material for: Depletion of Homeostatic Antibodies against Malondialdehyde-Modified Low-Density Lipoprotein Correlates with Adverse Events in Major Vascular Surgery
Source: Antioxidants (Basel). 2022 Jan 29;11(2):271. doi: 10.3390/antiox11020271 (PMC8868419; doi:10.3390/antiox11020271)
Supplement: Supplementary file 1 [file antioxidants-11-00271-s001.zip › antioxidants-1491451-supplementary.pdf]

Supplementary Tables

Supplementary Table S1. Primary and secondary endpoints of the Stress Induced Myocardial Infarction After Non-cardiac vascular surgery (SIMIAN) study, measured up to hospital discharge. *ECG: electrocardiogram.*

Supplementary Table 1

**Primary Endpoint**	**Secondary Endpoints**
Acute coronary syndrome determined by:	Unstable angina defined as:
*A rise in high sensitivity troponin T >60ng/L<br>AND<br>*Symptoms of ischaemia<br>OR<br>*New ECG changes<br>OR<br>*Development of pathological Q-waves	*Chest pain without significant troponin T rise<br>OR<br>*Permanent ECG changes with the development of new pathological Q-waves<br>OR<br>*T-wave inversion in two or more consecutive leads without symptoms
	Stroke defined as:<br>*Clinical diagnosis<br>OR<br>*Persisting neurological deficit lasting longer than 24 hours
	All cause mortality

Supplementary Table S2. Odds ratios of events (myocardial infarction, unstable angina or mortality) in relation to baseline levels of MDA-LDL, IgG/ IgM anti-MDA-LDL antibodies and related complexes. (Per SD increase in antibodies and in antibody tertiles). Model 1: adjusted for age and sex.

Supplementary Table 2

		Baseline Model 1	
		OR (95% CI)	p value
Per 1SD change in MDA-LDL		0.9 (0.55-1.48)	0.68
MDA-LDL	Low	1.00 (Ref)	
	Mid	1.24 (0.4-3.83)	0.7
	High	1.53 (0.52-4.49)	0.44
Trend			0.43
Per 1SD change in IgG anti-MDA-LDL		1.36 (0.87-2.15)	0.18
IgG anti-MDA-LDL	Low	1.00 (Ref)	
	Mid	2.35 (0.72-7.6)	0.15
	High	2.25 (0.7-7.26)	0.18
Trend			0.19
Per 1SD change in IgM anti-MDA-LDL		0.82 (0.53-1.27)	0.37
IgM anti-MDA-LDL	Low	1.00 (Ref)	
	Mid	0.65 (0.22-1.91)	0.43
	High	0.78 (0.27-2.23)	0.64
Trend			0.62
Per 1SD change in IgG/MDA-LDL complexes		0.79 (0.5-1.24)	0.3
IgG/MDA-LDL complexes	Low	1.00 (Ref)	
	Mid	0.79 (0.29-2.16)	0.65
	High	0.39 (0.12-1.25)	0.11
Trend			0.12
Per 1SD increase in IgM/MDA-LDL complexes		0.97 (0.62-1.51)	0.9
IgM/MDA-LDL complexes	Low	1.00 (Ref)	
	Mid	0.67(0.23-2.01)	0.48
	High	0.77 (0.27-2.22)	0.63
Trend			0.63

Supplementary Table S3. Odds ratios of events (myocardial infarction, unstable angina or mortality) in relation to baseline levels of total IgG/ IgM, C3 and C3/MDA-LDL complexes. (Per SD increase in antibodies and in antibody tertiles). Model 1: adjusted for age and sex.

Supplementary Table 3

		Baseline<br>Model 1	
		OR (95% CI)	p value
Per 1SD change in total IgG		1.04 (0.67-1.61)	0.86
Total IgG	Low	1.00 (Ref)	
	Mid	2.21 (0.73-6.64)	0.16
	High	1.37 (0.43-4.38)	0.6
Trend			0.62
Per 1SD change in total IgM		0.86 (0.56-1.34)	0.51
Total IgM	Low	1.00 (Ref)	
	Mid	1.77 (0.64-4.91)	0.27
	High	0.6 (0.18-2)	0.4
Trend			0.45
Per 1SD change in complement C3		1.38 (0.87-2.19)	0.18
Complement C3	Low	1.00 (Ref)	
	Mid	3.67 (1.05-12.82)	**0.04**
	High	3.11 (0.89-10.88)	0.08
Trend			0.09
Per 1SD change in C3/MDA-LDL complexes		1.16 (0.77-1.74)	0.49
C3/MDA-LDL complexes	Low	1.00 (Ref)	
	Mid	2.06 (0.62-6.82)	0.24
	High	2.56 (0.8-8.16)	0.11
Trend			0.12
